# Survival of umbilicus on a superiorly based flap after fleur-de-lis abdominoplasty: A case report

**DOI:** 10.1097/MD.0000000000029115

**Published:** 2022-05-20

**Authors:** Demetris Savva, Giulio Nittari, Filippo Gibelli, Andreas Vassiliou

**Affiliations:** a Plastic Reconstructive and Aesthetic Surgery, Nicosia General Hospital, Nicosia, Cyprus; b Telemedicine and Telepharmacy Centre, School of Medicinal and Health Products Sciences, University of Camerino, Camerino, Italy; c Section of Legal Medicine, School of Law, University of Camerino, Camerino, Italy.

**Keywords:** fleur-de-lis abdominoplasty, massive weight loss, umbilicus

## Abstract

**Introduction:**

Massive weight loss patients have a midline excess of abdominal adipose and skin tissue that contributes to an increased abdominal girth. This excess of tissue in these patients is not resolved with traditional techniques of abdominoplasty and usually the fleur-de-lis abdominoplasty technique is employed.

**Patient concerns:**

A 22-year-old male patient came to our clinic after a massive weight loss of 170 kg, requesting an abdominoplasty for the excess adipose and skin tissue.

**Diagnosis:**

Massive weight loss patient, with excess of adipose and skin tissue in the midline abdominal area.

**Interventions:**

Fleur-de-lis abdominoplasty technique was employed for treatment of massive weight loss.

**Outcomes:**

During the surgery, it was decided that the umbilicus blood supply via the inferior epigastric artery and median umbilical ligament needed to be ligated, to remove more tissue for better aesthetic result. The umbilicus survived on the collateral blood supply from ligamentum teres and superior epigastric collaterals.

**Conclusion/Lessons:**

In this case report we review our experience treating a massive weight loss patient using a fleur-de-lis abdominoplasty technique without preserving the umbilicus blood supply via the inferior epigastric artery and median umbilical ligament. We eventually relied on the collateral blood supply from ligamentum teres and superior epigastric collaterals, something that proved advantageous both in the survival of the umbilicus on the long run despite cutting off the main blood supply, and, the removal of further excess adipocutaneous tissue for a better aesthetic outcome.

## 1 Introduction

Massive weight loss patients have a midline excess of abdominal adipose and skin tissue that contributes to an increased abdominal girth. This excess of tissue is not resolved with traditional techniques of abdominoplasty, and usually the fleur-de-lis abdominoplasty technique is employed in these cases. What's more, umbilicus transposition is necessary after excessive abdominoplasties. Kelly was the first to describe the abdominoplasty technique using a transverse incision in 1899.^[1]^ Since then, many different variations of the procedure have been described providing better results, both functional and aesthetic-wise. Castañares^[2]^ was the first to describe a vertical incision (fleur-de-lis pattern) for improving both the horizontal as well as the vertical laxity in massive weight loss patients. An inverted V pattern excision with rounded peak on the upper abdomen can treat the circumferential component in these cases with good results.^[3]^

Usually in such cases the supply to the umbilicus is preserved in a stalk and a transposition on the skin is performed to respect the anatomical markings on the outcome. The umbilicus blood supply is mainly through axial vessels from the inferior epigastric artery.^[4]^

In this case report we review our experience treating a massive weight loss patient using a fleur-de-lis abdominoplasty technique but without preserving the umbilicus blood supply via the inferior epigastric artery supply (and median umbilical ligament).^[5]^

## 2 Case presentation

**Figure 1:**
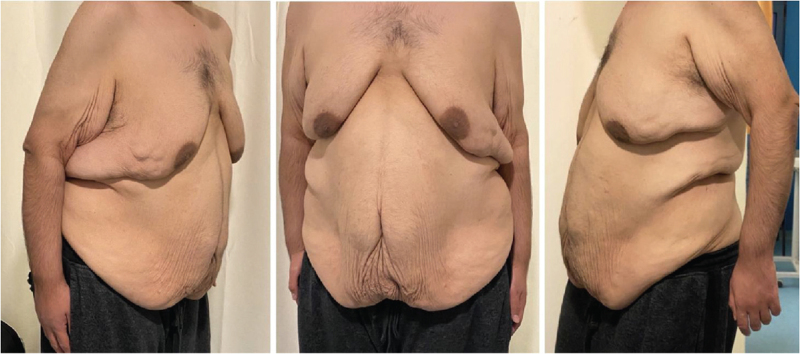
Photos of patient after massive weight loss, showing the excess tissue concerning the patient, especially in the abdominal area.

A 22-year-old caucasian, male patient came to our clinic after a massive weight loss of 170 kg, and stable weight for more than 1 year, complaining of excess abdominal adipocutaneous tissue despite his attempts to loose more weight (Fig. 1: *Photos after massive weight loss).* Initial body weight of the patient was 275 kg and through diet and exercise, there has been a BMI decrease of 55.5 units. The patient did not have any other medical comorbidities or previous operations undertaken.

Pre-operative measurements were taken of umbilical waist circumference (139 cm) and hip circumference (153 cm) in standing position. The patient was also marked in a standing position. The markings were used as a reference for the excision during surgery, but modifications were made in surgery to have the best possible result.

First, the inferior incision was marked from the superior border of the umbilicus to a curved line between the 2 superior anterior iliac spines.^[6]^ The fleur-de-lis pattern was completed with an inverted V-marking, with rounding of the peak, in order to minimize the abdominal girth.

The patient was operated under general anesthesia, a Foley catheter was inserted, anti-thrombotic stockings were used and a round of prophylactic antibiotics was administered pre-operatively.

**Figure 2:**
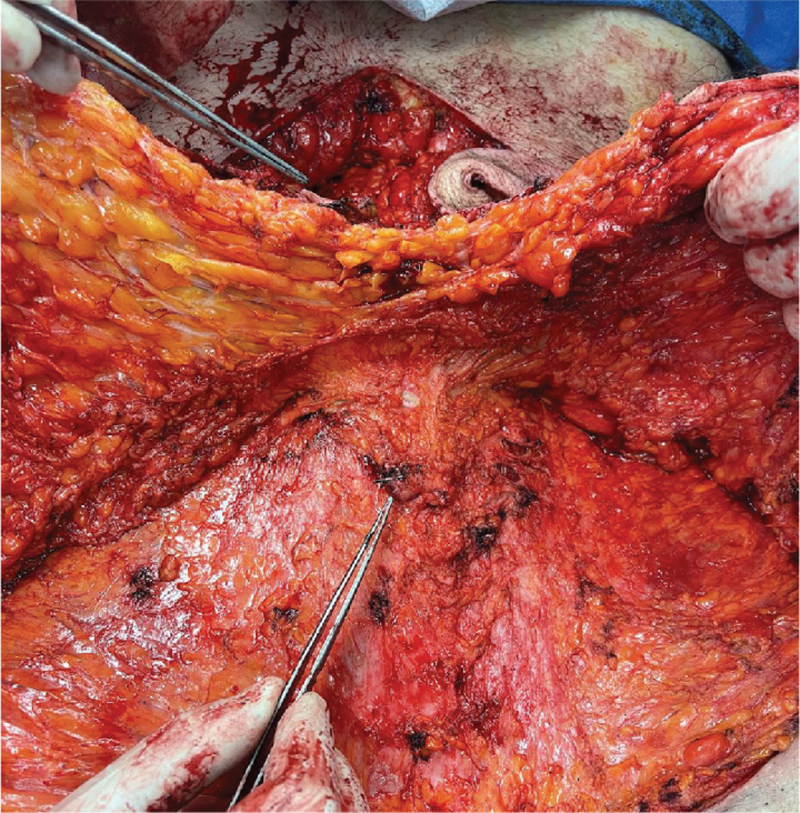
Location of ligated umbilical stump, before dissecting more superiorly.

**Figure 3:**
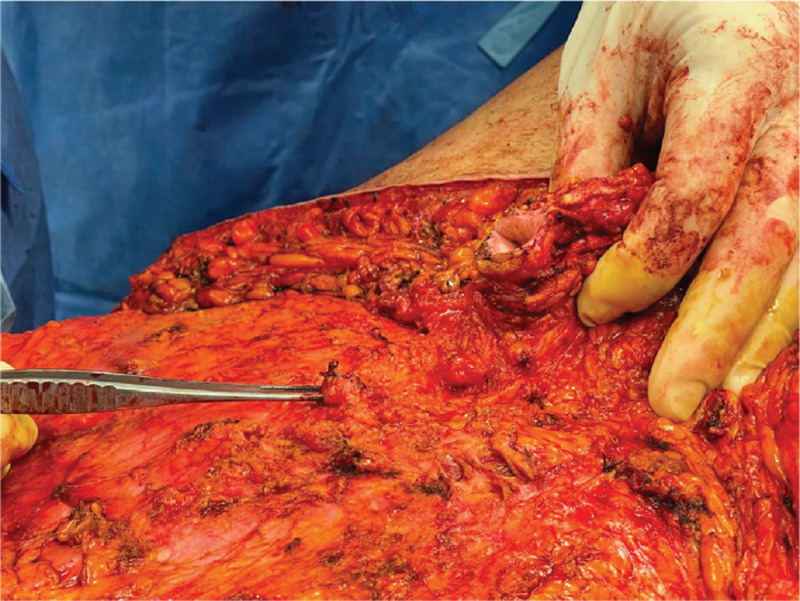
Ligated umbilical stump; location of ligated stump compared to the umbilicus position.

The lower incision was done up to the muscle fascia. Any bleeding point was cauterized. The tissue was elevated to the superior umbilical border. The incisions on the vertical plane were done using the xyphoid process centrally and the costal margins laterally as reference points. The flap was undermined up to the level of the anterior rectus sheath. Due to the difficulty preserving the umbilical stump with the median umbilical ligament because of the massive amount that needed to be disposed, we decided intrα-operatively to ligate it and rely on the collateral blood supply of the umbilicus. (Fig. 2: *Location of ligated umbilical stump;*Fig. 3: *Ligated umbilical stump).* Such an approach is not described in traditional fleur-de-lys abdominoplasties, but we decided to proceed due to the benefits that this modification provided.

**Figure 4:**
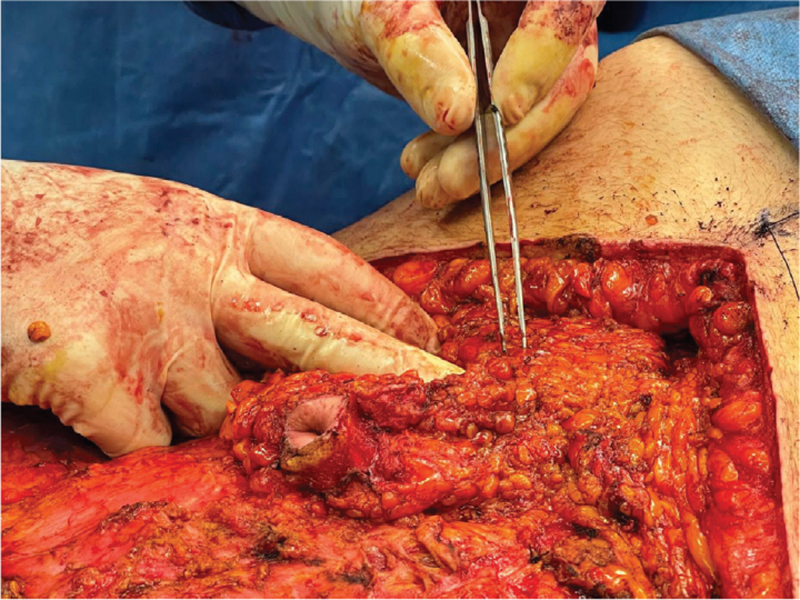
Superior pedicle of umbilicus before undermining, including the ligamentum teres and branches of the superior epigastric artery.

**Figure 5:**
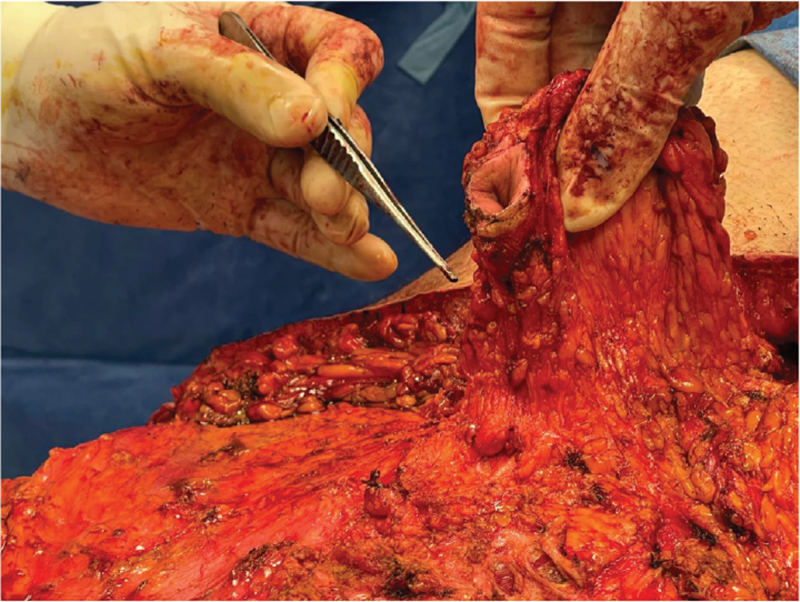
Umbilicus attached on remaining superior pedicle.

Therefore, a superior pedicle was preserved from the superior border of the umbilicus to the xyphoid process that included the ligamentum teres and branches of the superior epigastric artery (Fig. 4: *Superior pedicle before undermining;*Fig. 5*: Umbilicus attached on remaining superior pedicle).*

The excess tissue was removed, and the markings were checked to meet at the lower midline marking. Plication of the rectus fascia was performed to enhance the narrowing of the waistline with Polydiaxone-0 sutures. Two closed suction drains were placed in the mons pubis area and closure by layer was performed. The umbilicus was stabilized in a new rhomboid incision at its new position with Monofilament 3-0 sutures. Steri-strips were placed along the suture lines and an abdominal binder was placed.

The total tissue removed was 4.3 kg, weighted at the end of the operation. Intrα-operative blood loss was minimal thus no transfusion was necessary, and the total operation time was two and a half hours.

**Figure 6:**
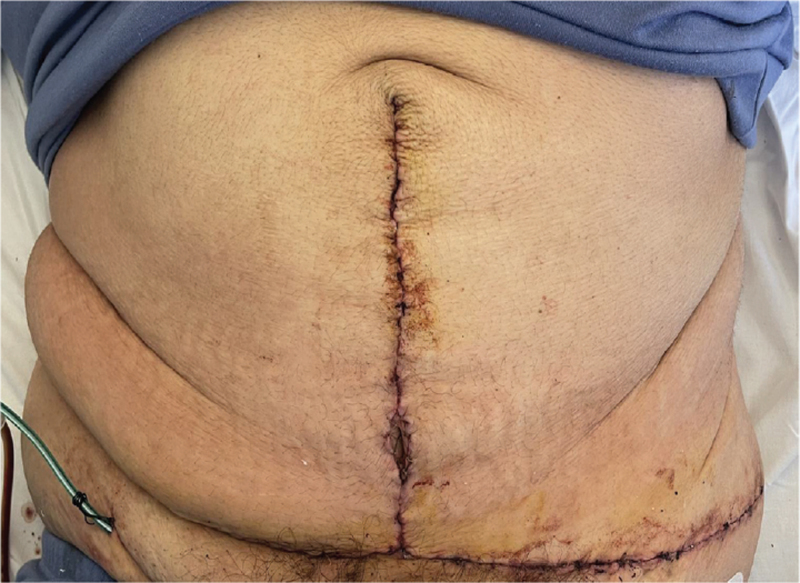
Photo 24 h postoperatively, showing incision wound area and drains.

The patient remained in the hospital for 2 days post- operatively, under analgesia and post-operative antibiotic use, minimal movements and 24/7 use of the binder (Fig. 6: *Photo 24 hours postoperatively).* Drains were removed after a 48-hour period, since their outcome was < 20 ml/24 h. Daily changes were done at the wound incision lines and checks for the viability of the umbilicus. No post-operative complications were noted.

**Figure 7:**
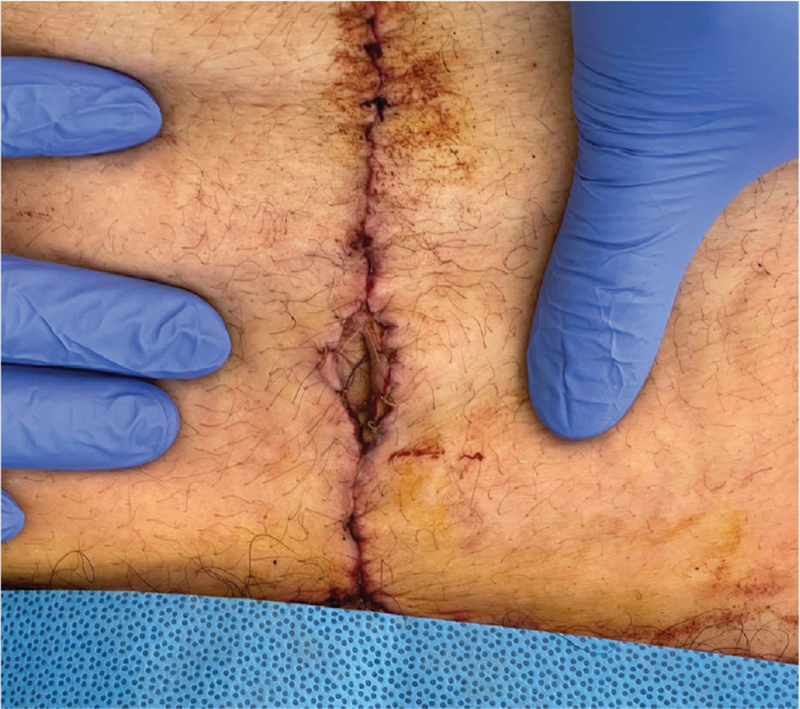
Photo 1 wk postoperatively, showing no complications with the umbilicus.

**Figure 8:**
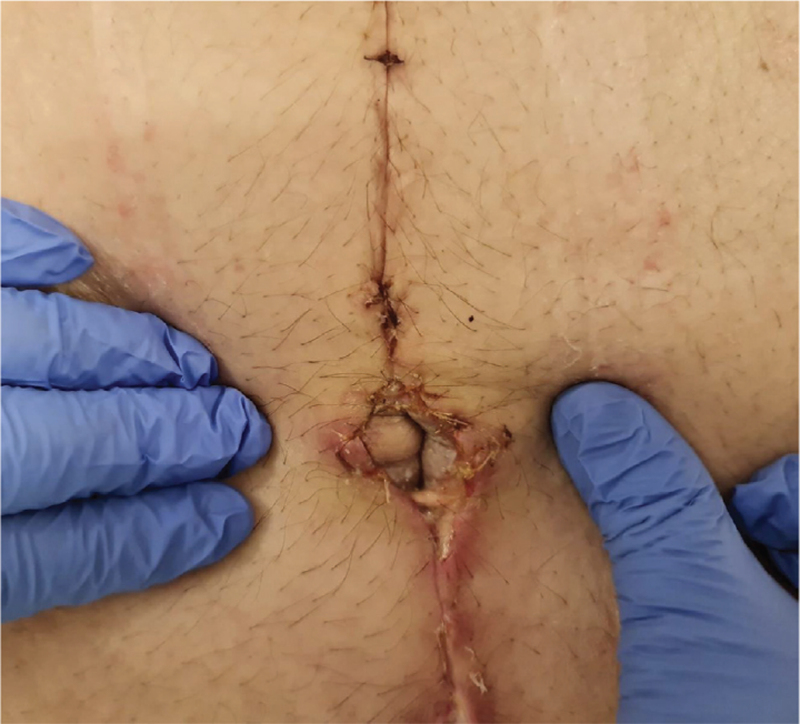
Photo 1 mo postoperatively; survival of umbilicus with no complications and healed incision wounds.

The patient was discharged with instructions to wear the abdominal binder for the next month continuously, regular wound changes, avoid exercise and weightlifting for at least 4 weeks and analgesia medication when needed. The patient was followed-up on a 1-week, 2-week, 1-month, and 3-month intervals (Fig. 7: *Photo 1 week postoperatively;*Fig. 8: *Photo 1 month postoperatively).*

Post-operative measurements were taken at 3 months, with umbilical waist circumference down to 121 cm, and hip circumference to 130 cm.

## 3 Discussion

Extensive abdominoplasty techniques need to be employed in cases of massive weight loss. The fleur-de-lis pattern is a very good approach for patients with massive weight loss since it has the advantage to compensate for the abdominal girth component, not treated with simple, single incision abdominoplasties. Traditionally, the umbilical stump is preserved while transpositioning. The disadvantage in the traditional fleur-de-lis technique is the limitation in the tissue excised due to the stump presence in the lower surgical field. Recent reports in literature describe sacrifice and removal of umbilicus, usually in cases of a particularly large pannus and elongating the umbilicus is not likely to survive due to vascular insufficiency.^[7]^

In our case report, we proved that collateral blood supply to the umbilicus can be successfully used to preserve its viability in difficult cases where the inferior epigastric artery supply needs to be interrupted.

Candidates for this modification technique can therefore include large abdominal girth pannus patients, or when the inferior epigastric artery supply to the umbilicus need to be interrupted, without the need of sacrificing the umbilicus. This is of vital importance in cases like this one, where more tissue needs to be excised to provide a better aesthetic outcome. Cosmetic surgeons can therefore proceed safely with further resection, without the need to preserve the main, inferior epigastric artery supply, since the umbilicus can survive on the collateral blood supply from ligamentum teres and superior epigastric collaterals.

## Author contributions

**Conceptualization:** Demetris Savva, Andreas Vassiliou, Giulio Nittari.

**Data curation:** Filippo Gibelli.

**Methodology:** Andreas Vassiliou.

Project administration: Demetris Savva.

**Supervision:** Andreas Vassiliou.

**Writing** – **original draft:** Demetris Savva, Filippo Gibelli.

Writing – review & editing: Giulio Nittari.
